# Learning curve of surgical novices using the single-port platform SymphonX: minimizing OR trauma to only one 15-mm incision

**DOI:** 10.1007/s00464-020-07998-3

**Published:** 2020-09-23

**Authors:** Rabi R. Datta, Sebastian Schönhage, Thomas Dratsch, Justus Toader, Dolores T. Müller, Roger Wahba, Robert Kleinert, Michael Thomas, Georg Dieplinger, Dirk L. Stippel, Christiane J. Bruns, Hans F. Fuchs

**Affiliations:** Department of Surgery, University of Cologne, Kerpener Str. 62, 50937 Cologne, Germany

**Keywords:** Single-port surgery, SILS, Robotic surgery, Novices, Learning curve

## Abstract

**Background:**

Minimally invasive single-port surgery is always associated with large incisions up to 2–3 cm, complicated handling due to the lack of triangulation, and instrument crossing. The aim of this prospective study was to report how medical students without any laparoscopic experience perform several laparoscopic tasks (rope pass, paper cut, peg transfer, recapping, and needle threading) with the new SymphonX single-port platform and to examine the learning curves in comparison to the laparoscopic multi-port technique.

**Methods:**

A set of 5 laparoscopic skill tests (Rope Pass, Paper cut, Peg Transfer, Recapping, Needle Thread) were performed with 3 repetitions. Medical students performed all tests with both standard laparoscopic instruments and the new platform. Time and errors were recorded.

**Results:**

A total of 114 medical students (61 females) with a median age of 23 years completed the study. All subjects were able to perform the skill tests with both standard laparoscopic multi-port and the single-port laparoscopic system and were able to significantly improve their performance over the three trials for all five tasks—rope pass (*p* < 0.001), paper cut (*p* < 0.001), peg transfer (*p* < 0.001), needle threading (*p* < 0.001), and recapping (*p* < 0.001). In 3 out of 5 tasks, medical students performed the tasks faster using the standard multi-port system—rope pass (*p* < 0.001), paper cut (*p* < 0.001), and peg transfer (*p* < 0.001). In the task recapping, medical students performed the task faster using the new single-port system (*p* = 0.003). In the task needle threading, there was no significant difference between the standard multi-port system and the new single-port system (*p* > 0.05).

**Conclusion:**

This is the first study analyzing learning curves of the commercially available SymphonX platform for abdominal laparoscopic surgery when used by novices. The learning curve and the error rate are promising.

Since its introduction to clinical practice laparoscopic surgery underwent an enormous development [1, 2]. The pathway of minimally invasive surgery until the most recent milestones such as single-port and robotic surgery has been reported before [3–12]. In this context, we recently described our first preclinical experience with a newly developed single-port device, fitting through a standard 15-mm trocar [3, 4]. More recently, we were able to demonstrate the feasibility of true single-port surgery using the in the meantime certified “SymphonX platform” performing the first clinical series of laparoscopic cholecystectomy through only one 15-mm trocar without any assisting instruments [11].

One of the main challenges of the single-port technique is the long learning curve. Due to missing triangulation, the use of various crossing instruments via one port has been a challenge for many users [13]. Standard laparoscopic multi-port techniques seem to be more intuitive in comparison to single-port procedures, but on the other hand not all laparoscopic skills appear to be transferable to the single-port technique [12, 14].

Various researchers have already described the learning curve for experienced laparoscopic surgeons gaining skills for single-port surgery, a fact that we were also able to show with our own data [11, 15, 16].

Introducing new surgical technology is a time-consuming process that needs to be handled with care for patients’ safety [17]. The aim of this study was to analyze the differences in usage of the new technology versus standard laparoscopy when performed by medical students without any laparoscopic experience.

## Material and methods

### Study design

In this single-center, prospective randomized study, medical students of the University Hospital Cologne without any previous experience in laparoscopic or single-incision surgery were recruited through posters, flyers, and email lists. All students who fit the selection criteria (medical students and no previous experience in laparoscopic or single-incision surgery) were offered inclusion in the study.

**Fig. 1:**
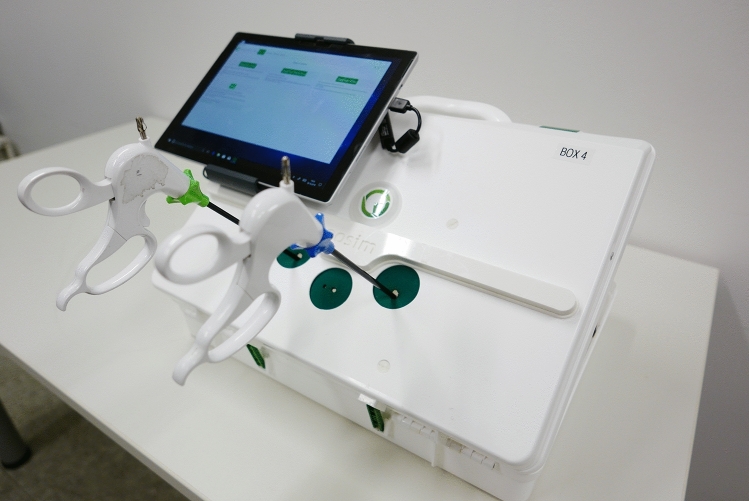
eoSim laparoscopy box

Laparoscopic skills were measured using two different laparoscopic training simulators: eoSim (eoSurgical Ltd, Edinburgh, UK), a commercially available simulation box in the form of a suitcase with three accesses, which basically allows the insertion of three instruments. A camera system is integrated, which transmits the images to the user via a tablet. At the same time, the tasks could be recorded using this system (Fig. 1).

**Fig. 2:**
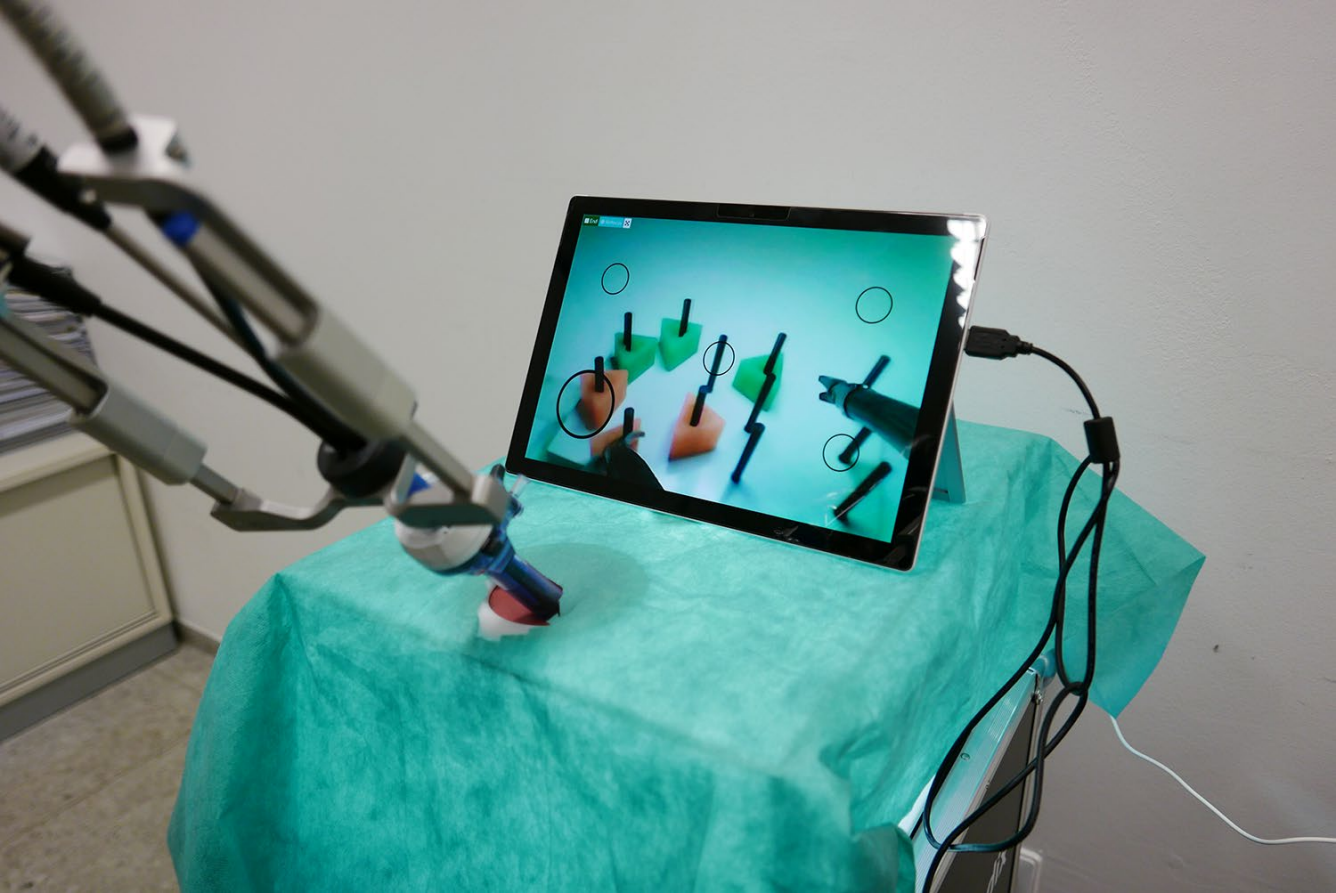
Fortimedix laparoscopic box, front view

**Fig. 3:**
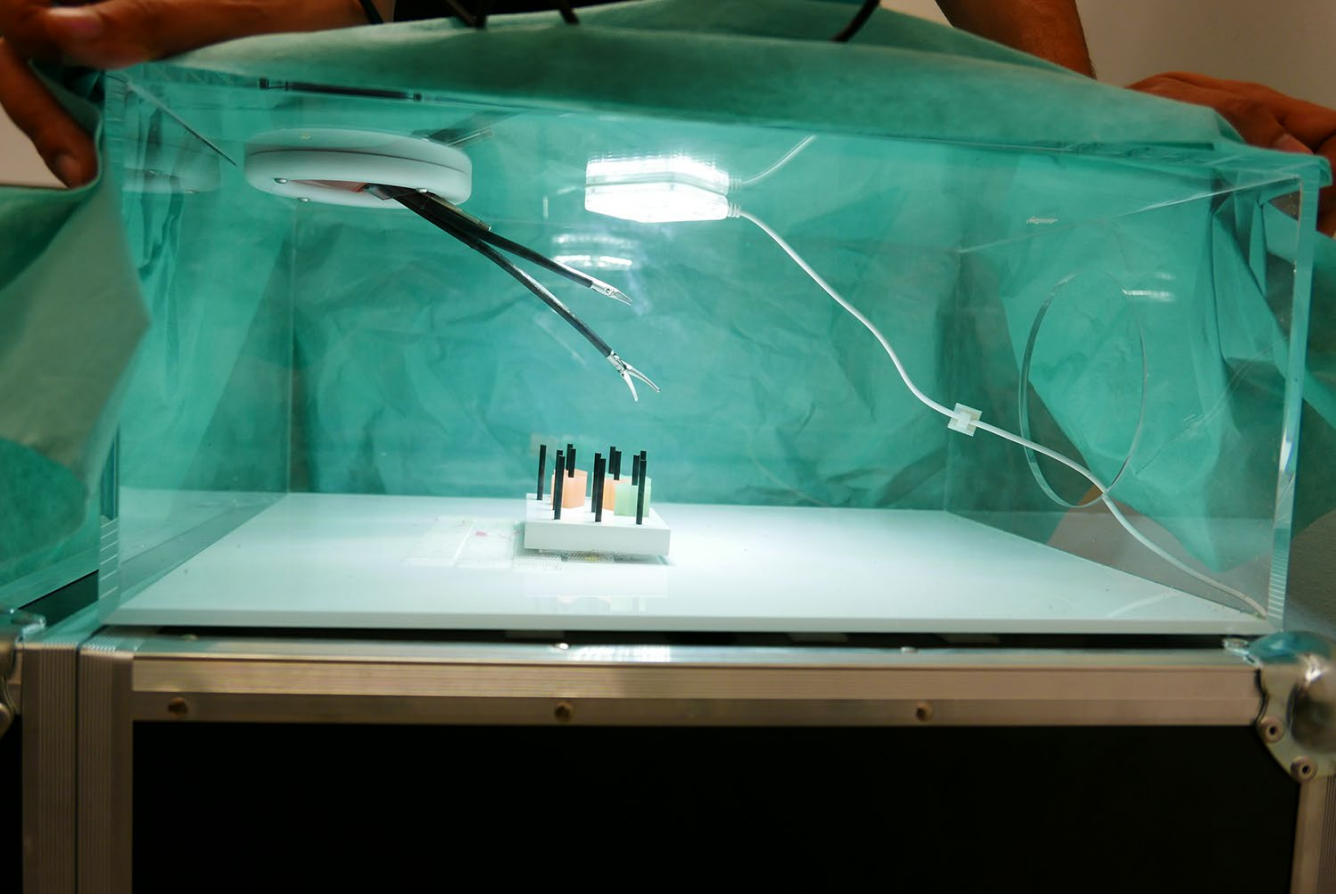
Fortimedix laparoscopic box, side view

The Fortimedix-simulation box (Fortimedix, Nuth, Netherlands.) was developed especially for this study to make the application as realistic as possible. The recording system was based on an endoscopic camera, being inserted into the device through the intended opening. The camera was always held by the instructor without interfering the exercises. The image was transmitted via a tablet (Figs. 2, 3).

At the beginning, students watched an instructional video explaining the five laparoscopic tasks. After that, students received a handout describing all five tasks in more detail. According to their randomized classification, the students were brought to their corresponding simulator. Before starting the exercises, the test person had the opportunity to become familiar with the respective instruments under the supervision and explanation of the instructor. As soon as this was the case, the exercises were started. The acclimatization time was never longer than 60 s. Students had to complete each laparoscopic task three times. All laparoscopic tasks were recorded on video. For each task, time to complete and number of errors was measured based on the videos. The beginning of each trial was defined as the moment when the students first touched the materials of the task at hand with the laparoscopic instruments. The end of each trial was defined as the moment when students had completed the task and had released the laparoscopic instruments onto the floor of the laparoscopic training box.

**Fig. 4:**
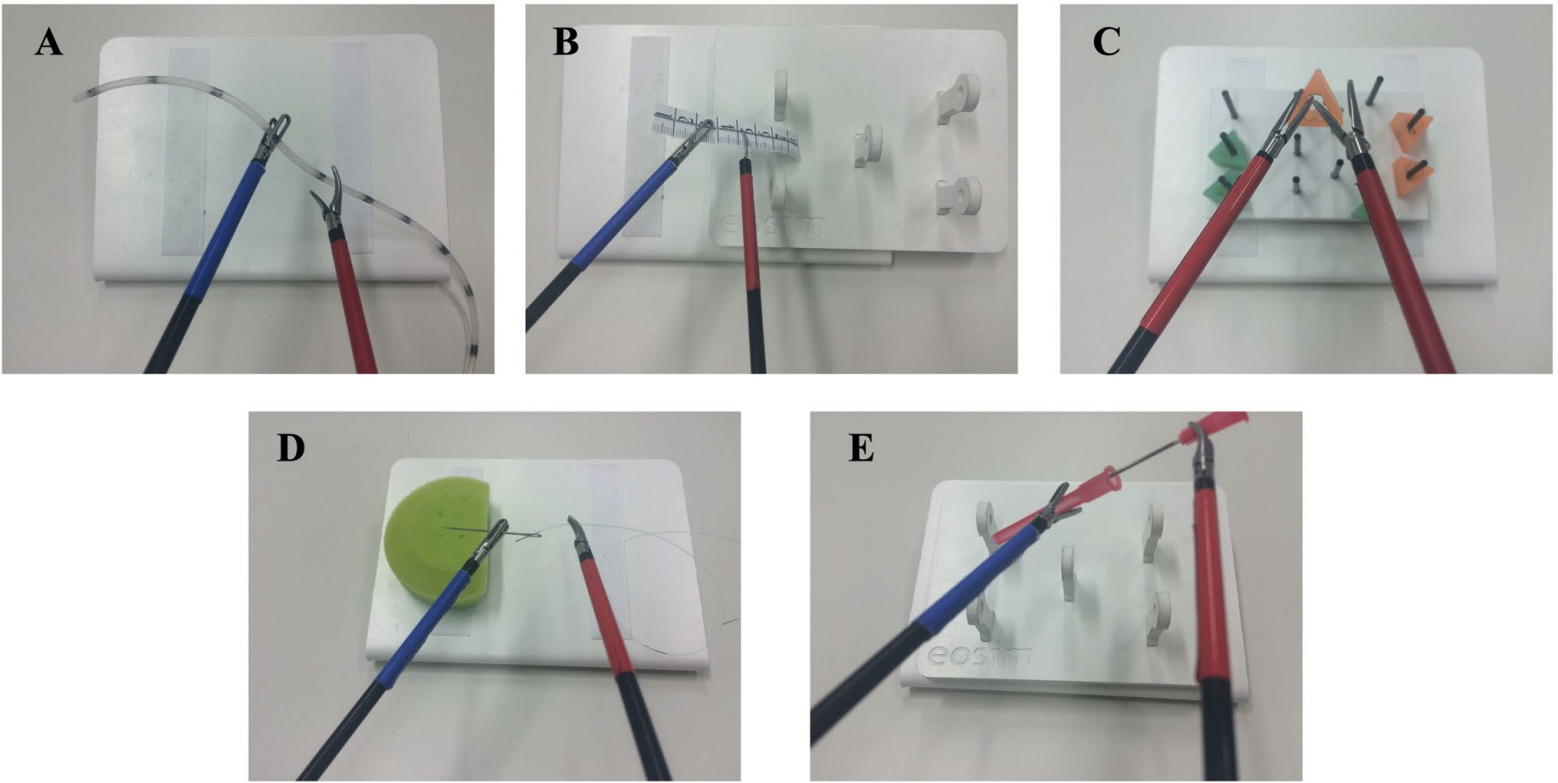
Tasks

The following laparoscopic tasks according to our previously published study protocol were used to measure laparoscopic skills: rope pass, paper cut, pegboard transfer, needle threading, and recapping [18]. These laparoscopic tasks were selected because they have been used as valid measurement tools in several prior studies [18–20]. The five tasks are depicted in Fig. 4.

In the rope pass task, the task was to pass a 30-cm-long silicone tube from one instrument to the other, while only touching the tube at certain marked areas (size of each area was 3 mm; the space between each area was 3 cm). Touching the silicone tube at a non-marked area was counted as an error.

In the paper cut task, students were presented with an 8-cm-long paper ruler with markings every millimeter and every centimeter. The task was to cut along the markings without fully cutting the paper ruler in half. Cutting through the paper or cutting in a non-marked area was counted as an error.

In the pegboard transfer task, students were presented with a pegboard with 11 metal rods and six triangles. The task was to transfer the triangles between the metal rods. Incorrect placement of the triangles was counted as an error.

In the needle threading task, students were presented with a needle sticking in a pincushion and thread that was marked in black on both ends and in the middle. The students were tasked with grabbing the needle with the left instrument and passing the thread through the eye of the needle, using the right instrument. Dropping the needle or the thread was counted as an error.

In the recapping task, students were presented with a needle and a cap. The cap was placed behind the lower left peg and the needle behind the right peg on the pegboard. The task was to grab both the needle and the cap using the instruments, recap the needle, and place the recapped needle behind the middle peg on the pegboard. Dropping the needle, the cap, or the recapped needle was counted as an error.

### Questionnaires

After participants had completed the laparoscopic tasks, they completed the NASA task load index (NASA-TLX) [21] as well as several questions about their prior gaming experience.

### Description of the new platform

**Fig. 5:**
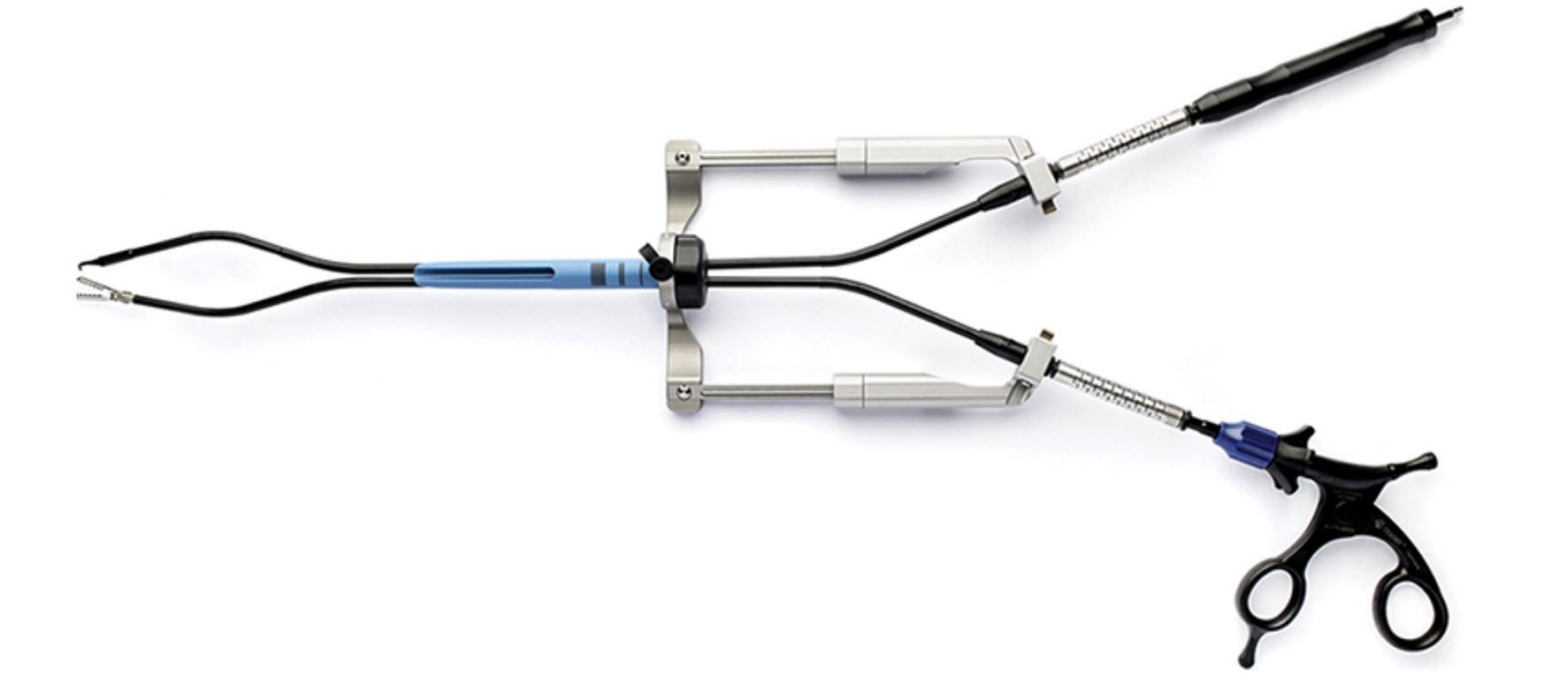
SymphonX platform

SymphonX includes an introducer fitting into a standard 15-mm trocar (Fig. 5). On each side of the introducer, lateral arms provide positional support of the articulating instruments. Instruments can be introduced via four lumens through the introducer: two lateral lumens for articulating instruments, a superior lumen for a 5-mm laparoscopic camera, and an inferior lumen for an additional instrument such as a 3-mm suction/irrigation device. Currently available instruments are hook cautery, alligator grasper, curved dissectors, clip applier, scissors, and suction/irrigation. In this study, we used only the curved dissectors and scissors. After insertion via the lateral lumens of the introducer, the devices are attached into the instrument clamps on the lateral guiding rails to activate a triangulated approach within the surgical field. The devices have special configurated segments which allow triangulation incorporating robotic features without proximal instrument crossing or collision. Each device is capable of 360° axial rotation, as well as lateral, anterior/posterior, and superior/inferior maneuverability. An instructional video of the technology can be seen in the supplementary material of this study.

### Procedure

Two students participated in each testing session. At the beginning of the experiment, students were greeted by the experimenter and sat down in front of a computer. Students then watched an instructional video describing the five laparoscopic tasks. After that, they received a handout describing the tasks. Then each student was assigned randomly to the laparoscopic box with the standard multi-port system or the single-port system box (Figs. 3, 4) and started with the first trial of the first task. Students always completed the five laparoscopic tasks in the following order: rope pass, paper cut, pegboard transfer, needle threading, and recapping. Each laparoscopic task was performed three times by each student. Due to the complexity of the needle threading and recapping tasks, a time limit of 15 min was set for both tasks. After 15 min had passed, students were asked to drop their instruments and move on to the next task. All laparoscopic tasks were recorded on video. After students had completed the laparoscopic tasks, they switched laparoscopic boxes. After students had completed the tasks on both laparoscopic systems, they completed several questions about their prior gaming experience and the NASA task load index (NASA-TLX).

### Participants

One hundred and fourteen medical students (53 males, 61 females; mean age = 23.3, age range 19–37) participated in the study. For detailed demographic information see Table 1. All students were recruited at the University Hospital of Cologne.

**Table 1:** Demographic data

	Male	Female
*n*	53	61
Age (Mean, SD)	23.5 (3.0)	23.2 (3.3)
Handedness (left:right)	4:49	4:57
Glasses (yes:no)	25:28	25:36
Semester		
2nd Preclinical	0	3
3rd Preclinical	4	4
4th Preclinical	7	8
1st Clinical	10	12
2nd Clinical	5	16
3rd Clinical	4	0
4th Clinical	4	5
5th Clinical	10	8
6th Clinical	1	0
Practical year	8	5

### Statistical analysis

For each of the three laparoscopic tasks, time to complete and number of errors were recorded. In line with Rosser et al., for each error 5 s were added to the time to complete the task [22]. This combined measurement was used in all statistical analyses. A statistical power analysis was performed for sample size estimation. Our study was sufficiently powered to detect medium-sized effects (Cohen’s *d* = 0.3) for the within-group comparisons. With an alpha = 0.05 and power = 0.80, the projected sample size needed to detect a medium effect for the within-group comparisons was *N* = 90 (GPower 3.1). With regard to correlations, a minimum sample size of *N* = 84 was needed to detect a medium-sized effect (GPower 3.1). Data were analyzed using the Statistical Package for the Social Sciences (SPSS, Version 25; IBM, 2017). Group comparisons were conducted using *t* tests and mixed ANOVAs. Kendall’s *τ* was used as a robust measure of correlation.

### Ethics

Ethics Committee approval was obtained before the study (Ethics Committee, University of Cologne) and the current study adheres to the criteria of our local ethics committee (No. 18-176). Written informed consent was given by all subjects before study inclusion.

## Results

### Rope pass

**Fig. 6:**
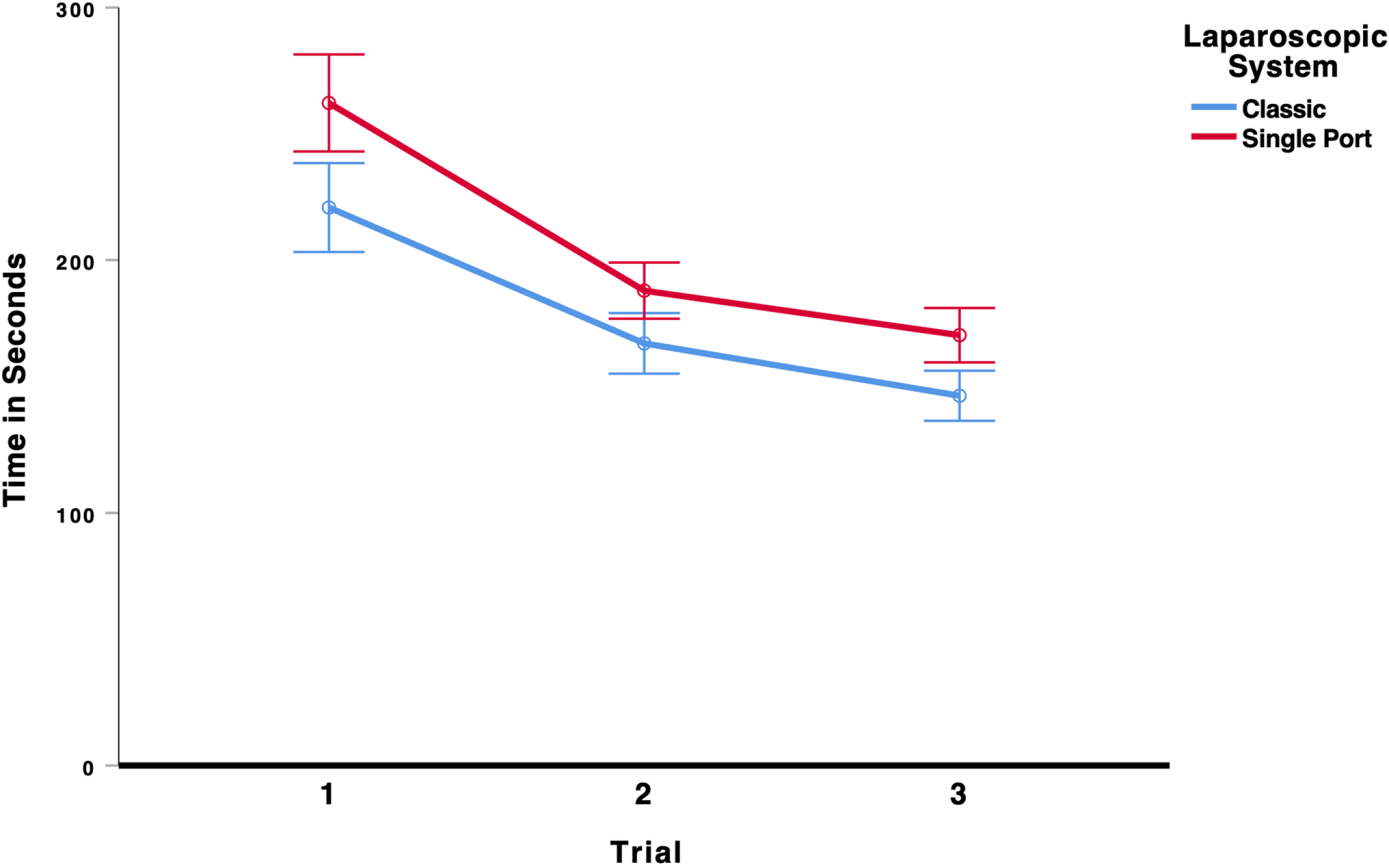
Time to complete the rope pass task. Error bars represent 95%-CI of the mean

To test whether laparoscopic performance improved over the three trials and whether the type of laparoscopic system (classic vs. single-port) influenced performance on the rope pass task, we conducted a 3 × 2 mixed ANOVA (Trial × Laparoscopic System). There was a significant main effect for trial, *F*(2,196) = 156.66, *p* < 0.001, *η*_p_^2^ = 0.615, indicating that students’ laparoscopic performance improved from the first to the third trial, see Fig. 1. There was also a significant main effect for laparoscopic system, *F*(1,98) = 15.31, *p* < 0.001, *η*_p_^2^ = 0.135, indicating that the students took on average longer to complete the rope pass task using the single-port system. All other effects were insignificant, all *F* < 2.55. As the results in Table 2 and Fig. 6 show, participants using the multi-port laparoscopic system did improve their performance from the first (221 s) to the last trial (146 s). Participants using the single-port system also improved their performance from the first (262 s) to the last trial (170 s), indicating that there was a significant learning curve effect for both systems.

**Table 2:** Mean time in seconds (95%-CI) to complete the task rope pass in each trial for the classic multi-port system and the new single-port system

Trial	Classic multi-port system			Single-port system		
	Mean	CI lower bound	CI upper bound	Mean	CI lower bound	CI upper bound
1	221	203	238	262	243	281
2	167	155	179	188	177	199
3	146	136	156	170	159	181

### Paper cut

**Fig. 7:**
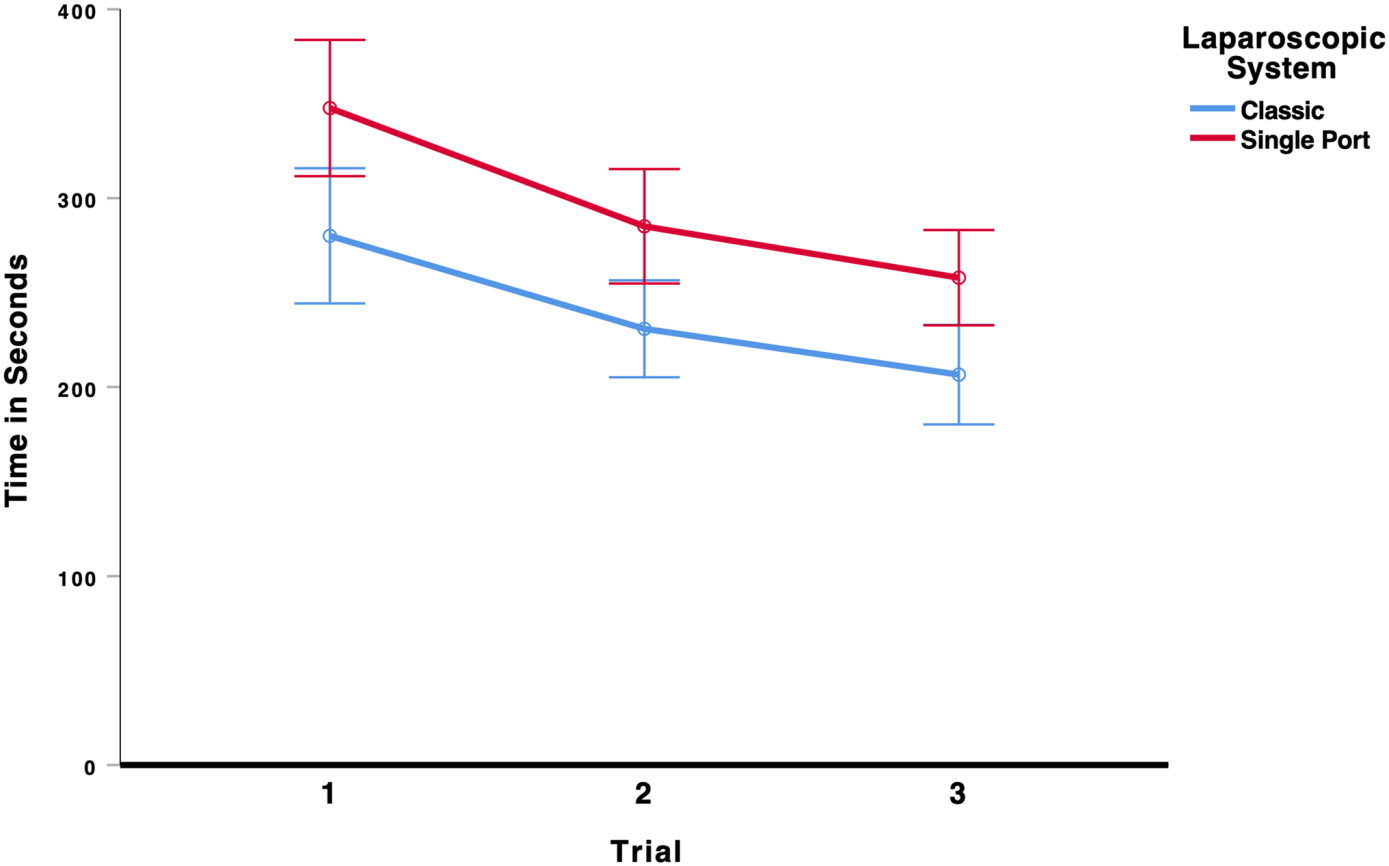
Time to complete the paper cut task. Error bars represent 95%-CI of the mean

To test whether laparoscopic performance improved over the three trials and whether the type of laparoscopic system (multi-port vs. single-port) influenced performance on the paper cut task, we conducted a 3 × 2 mixed ANOVA (Trial × Laparoscopic System). There was a significant main effect for trial, *F*(2,136) = 24.40, *p* < 0.001, *η*_p_^2^ = 0.264, indicating that students’ laparoscopic performance improved from the first to the third trial, see Fig. 7. There was also a significant main effect for laparoscopic system, *F*(1,68) = 17.52, *p* < 0.001, *η*_p_^2^ = 0.205, indicating that the students took on average longer to complete the paper cut task using the single-port system. All other effects were insignificant, all *F* < 0.25. As the results in Table 3 show, participants using the multi-port laparoscopic system did improve their performance from the first (280 s) to the last trial (207 s). Participants using the single-port system also improved their performance from the first (348 s) to the last trial (258 s), indicating that there was a significant learning curve effect for both systems.

**Table 3:** Mean time in seconds (95%-CI) to complete the task paper cut in each trial for the classic multi-port system and the new single-port system

Trial	Classic multi-port system			Single-port system		
	Mean	CI lower bound	CI upper bound	Mean	CI lower bound	CI upper bound
1	280	244	316	348	312	384
2	231	205	256	285	255	315
3	207	180	233	258	233	283

### Peg transfer

**Fig. 8:**
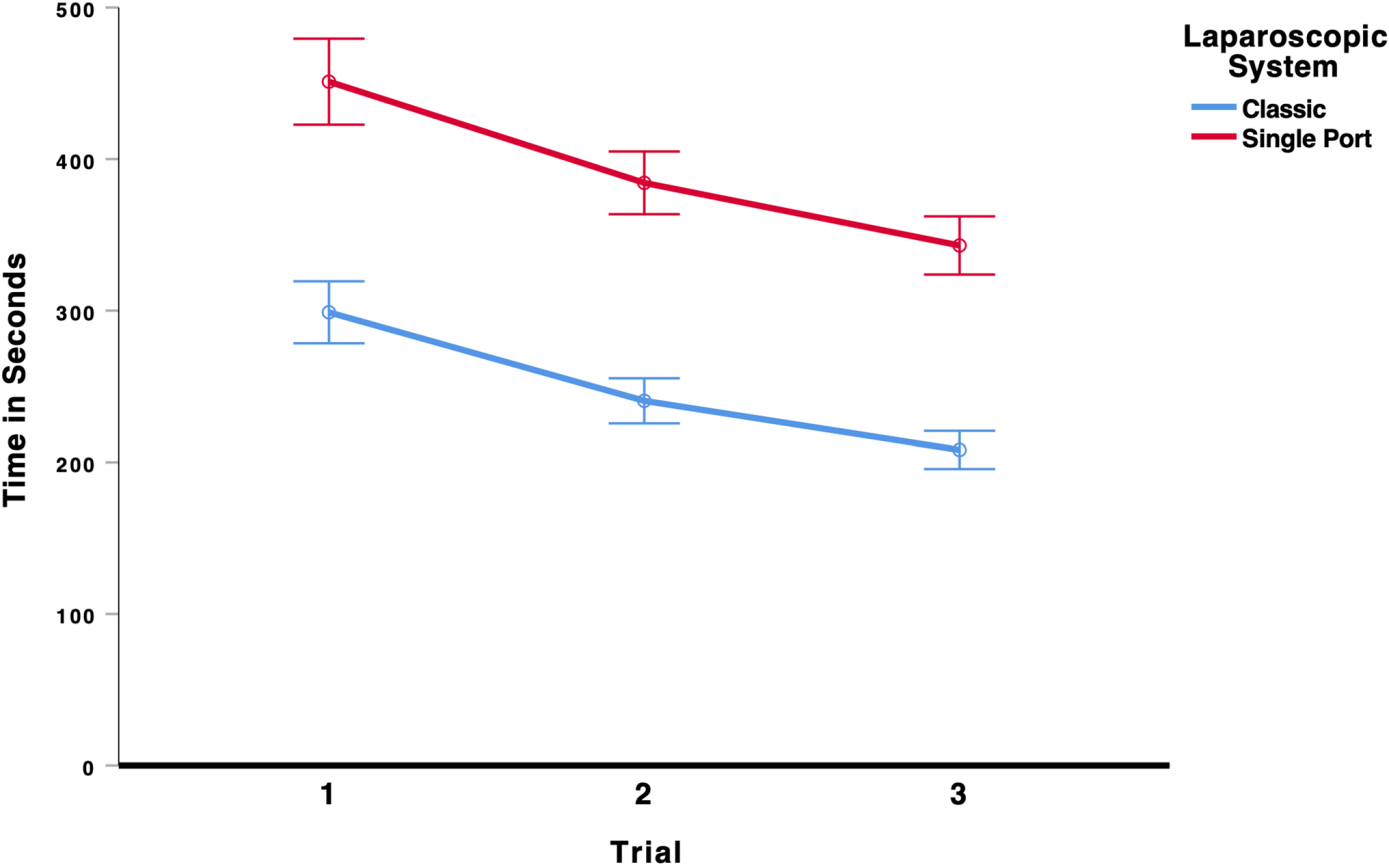
Time to complete the peg transfer task. Error bars represent 95%-CI of the mean

To test whether laparoscopic performance improved over the three trials and whether the type of laparoscopic system (multi-port vs. single-port) influenced performance on the peg transfer task, we conducted a 3 × 2 mixed ANOVA (Trial × Laparoscopic System). There was a significant main effect for trial, * F*(2,214) = 130.85, *p* < 0.001, *η*_p_^2^ = 0.550, indicating that students’ laparoscopic performance improved from the first to the third trial, see Fig. 8. There was also a significant main effect for laparoscopic system, *F*(1,107) = 262.80, *p* < 0.001, *η*_p_^2^ = 0.711, indicating that the students took on average longer to complete the peg transfer task using the single-port system. All other effects were insignificant, all *F* < 0.93. As the results in Table 4 show, participants using the multi-port laparoscopic system did improve their performance from the first (299 s) to the last trial (208 s). Participants using the single-port system also improved their performance from the first (451 s) to the last trial (343 s), indicating that there was a significant learning curve effect for both systems.

**Table 4:** Mean time in seconds (95%-CI) to complete the task peg transfer in each trial for the classic multi-port system and the new single-port system

Trial	Classic multi-port system			Single-port system		
	Mean	CI lower bound	CI upper bound	Mean	CI lower bound	CI upper bound
1	299	278	319	451	423	479
2	240	226	255	384	364	405
3	208	196	221	343	324	362

### Needle threading

**Fig. 9:**
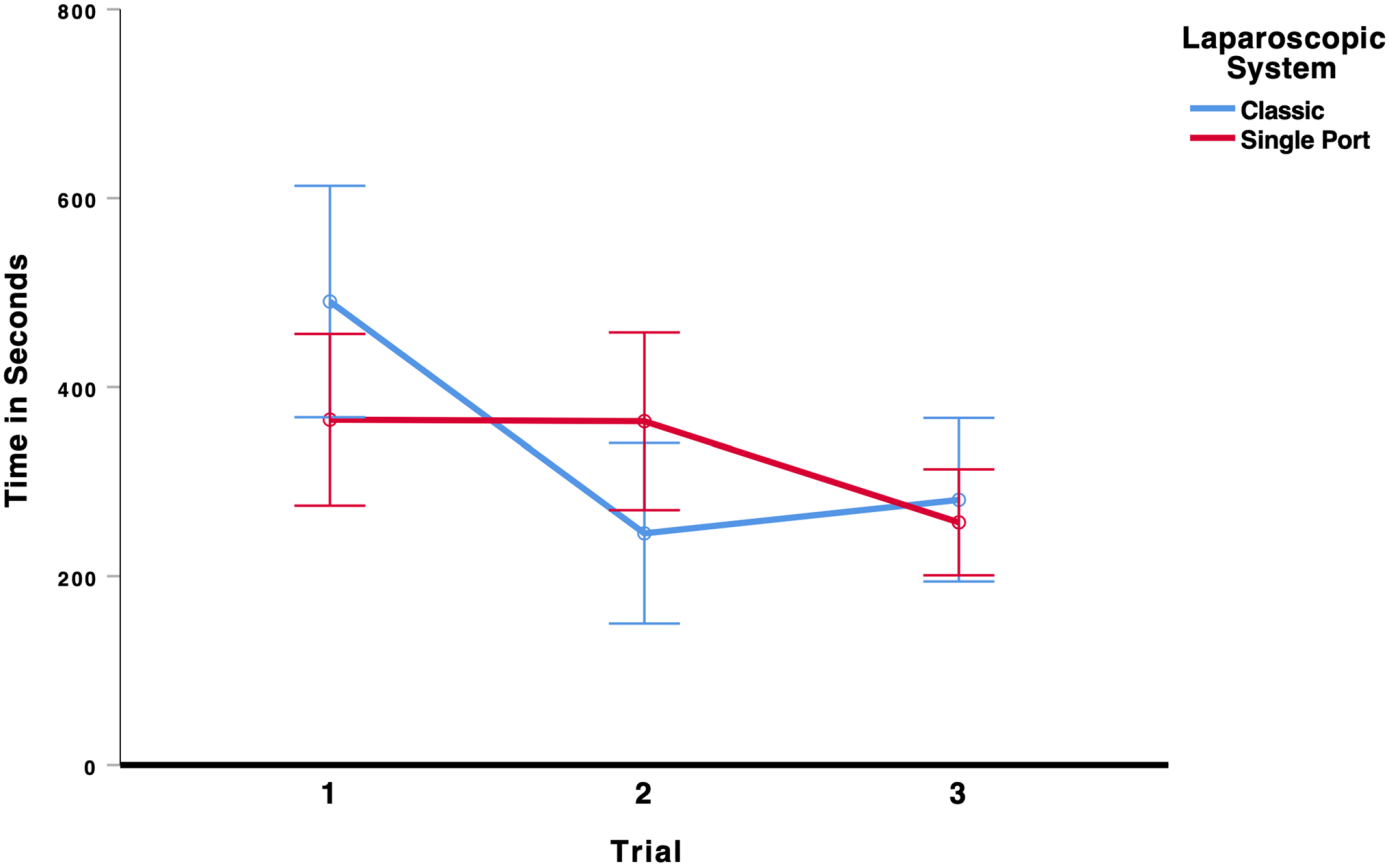
Time to complete the needle threading task. Error bars represent 95%-CI of the mean

To test whether laparoscopic performance improved over the three trials and whether the type of laparoscopic system (multi-port vs. single-port) influenced performance on the needle threading task, we conducted a 3 × 2 mixed ANOVA (Trial × Laparoscopic System). There was a significant main effect for trial, *F*(2,52) = 7.43, *p* < 0.001, *η*_p_^2^ = 0.222, indicating that the students’ laparoscopic performance improved from the first to the third trial, see Fig. 9. All other effects were insignificant, all *F* < 2.80. As the results in Table 5 show, participants using the multi-port laparoscopic system did improve their performance from the first (491 s) to the last trial (281 s). Participants using the single-port system also improved their performance from the first (365 s) to the last trial (257 s), indicating that there was a significant learning curve effect for both systems.

**Table 5:** Mean time in seconds (95%-CI) to complete the task needle threading in each trial for the classic multi-port system and the new single-port system

Trial	Classic multi-port system			Single-port system		
	Mean	CI lower bound	CI upper bound	Mean	CI lower bound	CI upper bound
1	491	368	613	365	275	456
2	245	150	341	364	270	458
3	281	194	367	257	201	313

### Recapping

**Fig. 10:**
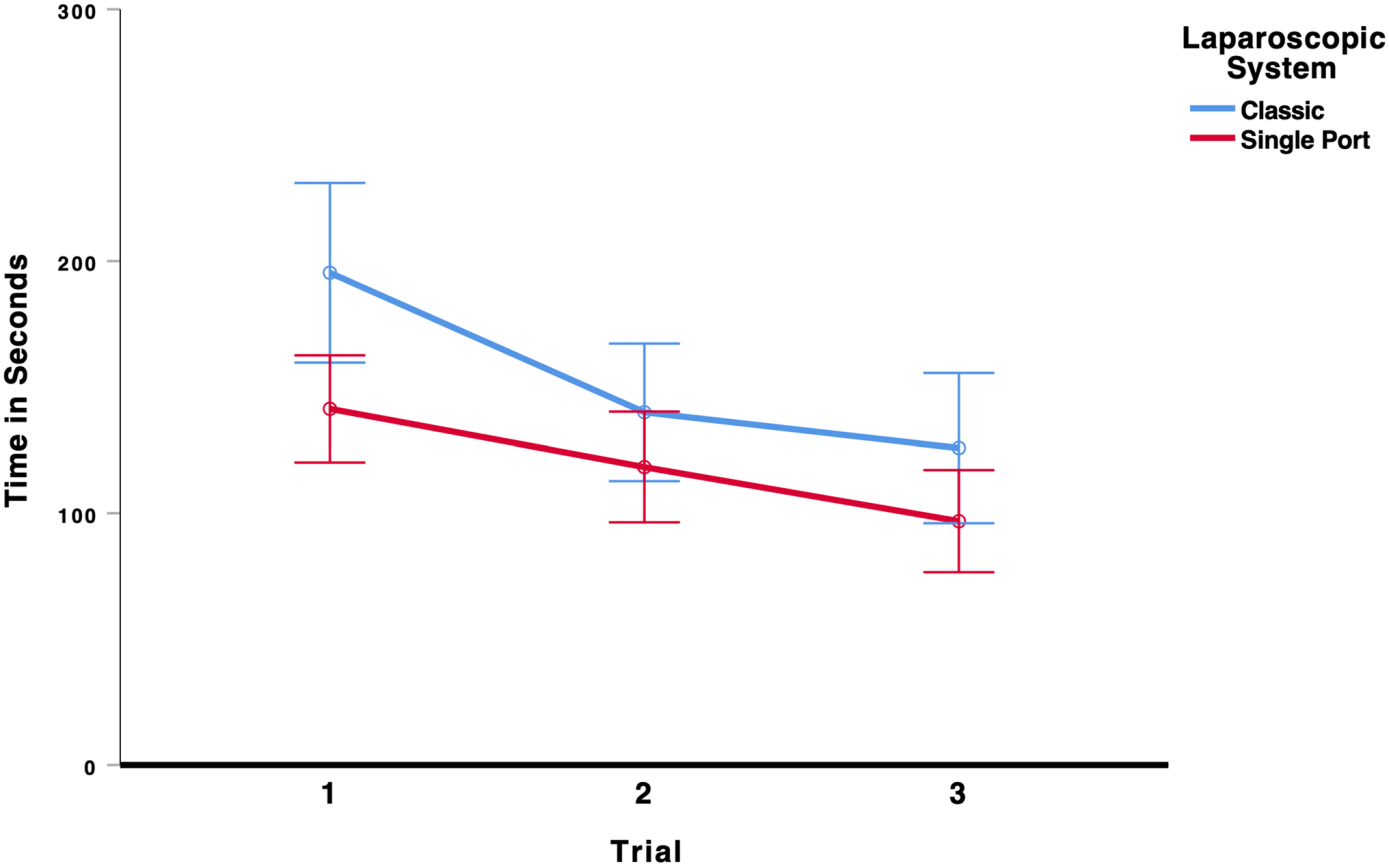
Time to complete the recapping task. Error bars represent 95%-CI of the mean

To test whether laparoscopic performance improved over the three trials and whether the type of laparoscopic system (multi-port vs. single-port) influenced performance on the recapping task, we conducted a 3 × 2 mixed ANOVA (Trial × Laparoscopic System). There was a significant main effect for trial, * F*(2,140) = 10.48, *p* < 0.001, *η*_p_^2^ = 0.130, indicating that students’ laparoscopic performance improved from the first to the third trial, see Fig. 10. There was also a significant main effect for laparoscopic system, *F*(1,70) = 9.70, *p* = 0.003, *η*_p_^2^ = 0.122, indicating that the students took on average longer to complete the recapping task using the classic system. All other effects were not significant, all *F* < 0.80. As the results in Table 6 show, participants using the multi-port laparoscopic system did improve their performance from the first (195 s) to the last trial (126 s). Participants using the single-port system also improved their performance from the first (141 s) to the last trial (97 s), indicating that there was a significant learning curve effect for both systems.

**Table 6:** Mean time in seconds (95%-CI) to complete the task recapping in each trial for the classic multi-port system and the new single-port system

Trial	Classic multi-port system			Single-port System		
	Mean	CI lower bound	CI upper bound	Mean	CI lower bound	CI upper bound
1	195	160	231	141	120	163
2	140	113	167	118	96	140
3	126	96	156	97	77	117

### Error rates

With regard to error rates, there was a significant difference between the laparoscopic systems (standard multi-port vs. single-port) for the tasks paper cut, *F*(1,68) = 92.91, *p* < 0.001, *η*_p_^2^ = 0.577, and recapping, *F*(1,70) = 13.95, *p* < 0.001, *η*_p_^2^ = 0.166. For the task paper cut, students made on average more errors using the single-port system. For the task recapping, on average students made more errors using the classic system. For the tasks rope pass and peg transfer, there was also the tendency for students to make more errors when using the single-port system. However, this difference was insignificant, all *F* < 3.82. For the task needle threading, there was a tendency for students to make more errors when using the standard multi-port laparoscopic system. However, this difference was insignificant, *F* < 4.19.

### Differences between laparoscopic systems on the NASA task load scale

To test whether students rated the two laparoscopic systems differently on the NASA task load scale, several paired *t* tests were performed. As the results in Table 7 show, students rated the mental, physical, and temporal demand to be significantly higher when using the single-port system. Additionally, effort to complete the tasks and frustration were also rated to be significantly higher when using the single-port system. In contrast, self-rated performance was significantly better when using the standard multi-port system.

**Table 7:** Differences between laparoscopic systems on the NASA task load scale

	Classic		Single-port		*t*	*p*	Cohen's d
	*M*	SD	*M*	SD			
Mental demand	4.15	2.22	5.09	2.43	5.272	0.001	0.502
Physical demand	4.34	1.95	6.05	2.19	8.217	0.001	0.781
Temporal demand	3.93	2.42	4.57	2.84	2.724	0.008	0.259
Effort	5.43	2.03	6.47	1.89	5.453	0.001	0.517
Performance^a^	3.87	2.16	4.50	2.18	3.488	0.001	0.334
Frustration	3.77	2.30	4.83	2.34	4.763	0.001	0.456

^a^This item was inversely coded. Lower values mean better performance

### Correlation of laparoscopic performance between the classic and single-port system

There was a significant correlation between the performance using the standard multi-port system and the single-port system, Kendall’s *τ*(112) = 0.176, *p* = 0.006. This suggests that students who performed better using one laparoscopic system also performed better using the other system.

### Correlation between laparoscopic performance and gaming questionnaire

As Table 8 shows, there was a significant negative correlation between measures of prior gaming experience and laparoscopic performance, indicating that students with more prior gaming experience performed the laparoscopic tasks quicker using both the classic and single-port system. However, more years of gaming experience lead to slower performance using the single-port system.

**Table 8:** Correlations (Kendall’s *τ*) between (overall) laparoscopic performance and gaming questionnaire (HGpD: Hours of gaming per day, DGpW: Days of gaming per week)

	Past HGpD	Past DGpW	Present HGpD	Present DGpW	Gaming Console	Playing Games	Years of Gaming
Classic	− 0.181*	− 0.298**	− 0.220**	− 0.248**	− 0.308**	− 0.223**	0.052
Single-Port	− 0.149*	− 0.159*	** − **0.103	− 0.159*	** − **0.084	− 0.158*	0.159*

*p < 0.05, **p < 0.01

### Preferred laparoscopic system

When asked what laparoscopic system they would like to use in the future, 64.9% (74 of 114) of the students stated that they preferred the standard multi-port system and 31.6% (35 of 114) of the students preferred the single-port system. 3.5% (4 of 114) reported no preference for any of the two systems.

### Other effects on performance

Wearing glasses had no significant effect on laparoscopic performance on either the standard multi-port or single-port system, all *t* < 0.67.

Students were categorized into three different groups (preclinical, clinical, and practical year) according to their level of clinical training. There was no significant effect of level of clinical training on laparoscopic performance on either the multi-port or single-port system, all *F* < 0.60.

## Discussion

This is the first study about surgical novices using the commercially available single-port surgical platform SymphonX that avoids large incision sizes and provides triangulation of instruments avoiding crossing.

Popularity of single-incision laparoscopic surgery increased in recent years and is utilized in many different surgical areas [23–30]. Difficulties in single-port surgery are mostly caused by impeded handling through straight and therefore crossing instruments. The SymphonX platform with its unique design differs from the majority of conventional single-port devices as it uses a standard 15-mm trocar and four non-crossing instruments. Furthermore, this device provides advanced triangulation in combination with a high range of motion, which makes it unique in comparison to other available single-port solutions mimicking robotic surgery on a technical level [11]. Therefore, results of previous studies concerning single-port devices may only be partially transferrable to the SymphonX platform.

In our previous studies, we demonstrated the feasibility of surgical procedures using the SymphonX platform [3, 4, 11]. This study now addresses, how laparoscopic novices, medical students without any laparoscopic experience, are able to deal with the new device. Every participant fulfilled each given task in both standard multi-port laparoscopy and using the single-port technique. One of the key messages is that there was a significant main effect for every trial, indicating improvement of the students’ laparoscopic performance from the first to the third trial, and it is astonishing that a relatively small number of repetitions led to a recognizable learning curve. A learning curve is defined as a longitudinal decrease and possible plateauing of different elements of a procedure [31]. For further evaluation, it is necessary to compare the platform with learning curves and other parameters of robotic and single-port operation systems; however, a comparable technology in the field of single-port surgery is currently not existent. Fransen et al. described a plateau phase after three repetitions of exercises performed using a conventional laparoscopic technique and single-port surgery [32]. However, various other studies described learning curves that differ from 5 to 75 cases for the use of single-port devices dependent on the complexity of the examined surgical procedure [31, 33–36].

The medical students in our study needed more time to complete the tasks using the new single-port device. However, the learning curves of the SymphonX platform were comparable to those using standard multi-port laparoscopy. All medical students performed the third trial significantly faster than the first trial using both the standard multi-port and the single-port system.

We demonstrated that a significant difference between the laparoscopic systems (standard multi-port vs. single-port) was seen for error rates. For the task paper cut, students made on average more errors using the single-port system. Besides that, a tendency for students making more errors for the tasks rope pass and peg transfer when using the single-port system, however without statistical significance, was seen. Interestingly, for the task recapping and needle threading, students made on average more errors using the standard multi-port laparoscopic system; however, differences also showed no statistical significance. As major triangulation is helpful for these exercises, SymphonX may have been superior here. Shakir et al. could demonstrate an advantage of robotics for novices performing the task recapping [37]. Here it was assumed that the advantages of robotics are more effective in more complex exercises like recapping. There is a constant debate whether single-port, standard multi-port, and robotic performances are transferable [11].

In our collective, a significant correlation between the performance using the classic laparoscopic system and the single-port system was found. Students who performed better using one laparoscopic system also performed better using the other, which also may allow for a “skills test” of novices. This may lead to a correlation between these two operation techniques and allows the presumption of a correlation between experience in conventional laparoscopic surgery and an easier start into single-port surgery.

When we asked the study participants which laparoscopic system they prefer for future use, a majority voted for the standard multi-port system. Struggling during the learning curve with a new surgical device often led surgeons to doubt the benefit and feasibility of new techniques and surgical systems like single-port devices [32]. The mental workload is also important to mention in this context. Many prior studies used the National Aeronautics and Space Administration Task Load index (NASA-TLX) score to measure the effect of procedures on surgeon’s workload [38–40]. In our cohort, students rated the mental, physical, and temporal demand to be significantly higher when using the single-port system. Additionally, the effort to complete the tasks and frustration were also rated and found to be significantly higher when using the single-port system. In contrast, self-rated performance was significantly better when using the standard multi-port system.

In line with our findings, the working group around Kim et al. was able to present similar results. Surgeons using a single-port system for cholecystectomy reached the lowest NASA-TLX scores in comparison to surgeons performing a laparoscopic cholecystectomy using three-port laparoscopic technique and surgeons performing a single-incision robotic cholecystectomy. Enumerated reasons were partly missing overview of anatomical structures, crossed instruments, and the single-port causing fatigue of surgeons through gas leakage [41]. This is relevant as an increased workload was associated with a poorer surgical performance [40]. Therefore, prior to implementation of new devices like SymphonX, intensive training sessions are necessary to avoid similar negative effects.

Interestingly, there was a significant negative correlation between measures of prior gaming experience and laparoscopic performance, indicating that students with more prior gaming experience performed laparoscopic tasks quicker using both the classic and the single-port system. This observation was shown in many other studies before [42–44].

Our study has several limitations. Although a learning curve was already shown after three repetitions, more runs may be necessary to draw serious conclusions. Also, more training time may be needed to experience the full advantages of the single-port platform. Not only novices, but also experts should be evaluated in future studies. Further studies also need to include more participants to increase the significance of the results.

## Conclusion

This is the first study of novices using the commercially available SymphonX platform. The learning curve of surgical novices using the new surgical platform SymphonX is comparable to standard multi-port laparoscopy in this series. Continued evolution and improvement of the device is ongoing to improve evidence of the new technology used. Future studies will focus on a comparison with other single-port devices.
